# PTEN in Colorectal Cancer: Shedding Light on Its Role as Predictor and Target

**DOI:** 10.3390/cancers11111765

**Published:** 2019-11-09

**Authors:** Lisa Salvatore, Maria Alessandra Calegari, Fotios Loupakis, Matteo Fassan, Brunella Di Stefano, Maria Bensi, Emilio Bria, Giampaolo Tortora

**Affiliations:** 1 Comprehensive Cancer Center, Fondazione Policlinico Universitario A. Gemelli IRCCS, 00168 Rome, Italy; mariaalessandra.calegari@gmail.com (M.A.C.); brunelladistefano89@gmail.com (B.D.S.); mariabensi@hotmail.it (M.B.); emilio.bria@policlinicogemelli.it (E.B.); giampaolo.tortora@policlinicogemelli.it (G.T.); 2 Medical Oncology, Universita’ Cattolica del Sacro Cuore, 00168 Rome, Italy; 3 Unit of Oncology 1, Department of Oncology, Veneto Institute of Oncology IOV – IRCCS, 35128 Padua, Italy; fotios.loupakis@iov.veneto.it; 4 Unit of Surgical Pathology, Department of Medicine, University of Padua, 35122 Padua, Italy matteo.fassan@gmail.com

**Keywords:** PTEN, colorectal cancer, target therapy, bevacizumab, anti-EGFR

## Abstract

Molecular assessment of colorectal cancer (CRC) is receiving growing attention, beyond RAS and BRAF, because of its influence on prognosis and prediction in cancer treatment. PTEN (phosphatase and tensin homologue), a tumor suppressor, regulating cell division and apoptosis, has been explored, and significant evidence suggests a role in cetuximab and panitumumab resistance linked to the epidermal growth factor receptor (EGFR) signal transduction pathway. Factors influencing PTEN activity should be analyzed to develop strategies to maximize the tumor suppressor role and to improve tumor response to cancer treatment. Therefore, an in-depth knowledge of the PI3K-Akt pathway—one of the major cancer survival pathways—and the role of PTEN—a major brake of this pathway—is essential in the era of precision medicine. The purpose of this literature review is to summarize the role of PTEN as a predictive factor and possible therapeutic target in CRC, focusing on ongoing studies and the possible implications in clinical practice.

## 1. Introduction

**Figure 1:**
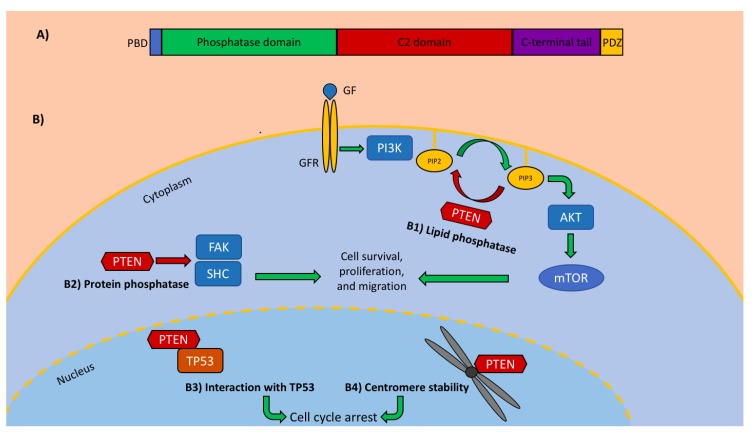
PTEN protein structure and functions. (**A**) PTEN structure. (**B**) PTEN functions. B1) Lipid phosphatase: PTEN dephosphorylates PIP3 to PIP2, inhibiting the PI3K/Akt signaling cascade. B2) Protein phosphatase: PTEN dephosphorylates protein substrates (including FAK and SHC), regulating cell migration and adhesion. B3) Interaction with TP53: via direct interaction with TP53, PTEN enhances TP53 stability and transcriptional activity, resulting in cell cycle arrest. B4) Centromere stability: via direct interaction with the centromere, PTEN preserves the chromosome stability. AKT: protein kinase B; FAK: focal adhesion kinase; GF: growth factor; GFR: growth factor receptor; mTOR: mammalian target of rapamycin; PBD: PIP2 binding domain; PI3K: phosphatidylinositol 3-kinase; PIP2: phosphatidyl-inositol-4,5-diphosphate; PIP3: phosphatidyl-inositol-3,4,5-triphosphate; PTEN: phosphatase and tensin homolog; SHC: Src homology 2 domain-containing protein; TP53: tumor protein p53.

*PTEN* (phosphatase and tensin homolog deleted on chromosome ten), first described in the late 90s, is a tumor suppressor gene located at 10q23 [1,2]. This gene encodes for a protein with five main functional domains: an N-terminal phosphatidyl-inositol-4,5-diphosphate (PIP2)-binding domain, a phosphatase domain, a membrane-targeting C2 domain, a C-terminal tail, and a PDZ binding motif (Figure 1A). PTEN is a multifunctional protein exerting biological activities, both dependently and independently of its catalytic phosphatase domain (Figure 1B). First of all, PTEN dephosphorylates phosphatidyl-inositol-3,4,5-triphosphate (PIP3), a lipidic product of phosphatidylinositol 3-kinase (PI3K). By removing one phosphate from PIP3, PTEN counteracts the PI3K/Akt signaling cascade, controls cell proliferation/invasiveness [3,4], and promotes apoptosis [5]. PTEN regulates cell migration, cell adhesion to surrounding tissues, and new blood vessel formation via dephosphorylation of protein substrates (FAK, SHC) [6]. Additionally, PTEN maintains the stability of cells’ genetic information through direct interaction with the tumor suppressor TP53 and centromeres [6].

All of these functions help to prevent uncontrolled cell growth, which can lead to tumor formation.

Loss of PTEN expression or function leads to persistent activation of the PI3K/Akt intracellular signaling cascade, which represents an oncogenic mechanism involved in colorectal carcinogenesis. During colorectal tumorigenesis, PTEN expression or function can be impaired at different levels: genomic, transcriptional, post-transcriptional, and post-translational [7]. In colorectal cancer (CRC), the loss of PTEN expression is estimated to occur in 34.5% of cases [8] and can result from both genetic and epigenetic mechanisms [9]. Genetic aberrations are rare events and include genomic mutations (2.02–13% in CRC with high microsatellite instability) [8,10,11] and decreased gene copy numbers (18.2–38.7%) [8,12].

Mechanisms silencing *PTEN* transcription are more frequent and are mainly represented by epigenetic promoter hypermethylation (27.3%) [8]. In addition, an even higher rate of protein loss of function due to post-translational modifications and altered protein–protein interaction or intracellular localization has been postulated [13].

This review aimed at defining an identikit of CRC-harboring PTEN alterations, assessing how these alterations predict a CRC-targeted treatment response that may be exploited in the future as effective target of innovative treatments.

## 2. PTEN in CRC

Several studies have demonstrated that PTEN alterations are associated with a specific clinicopathologic and molecular profile in CRC.

Day et al. screened 1093 patients with stage I–IV CRC for *PIK3CA* (exons 9 and 20), *KRAS* (codons 12–13), and *BRAF* (codon 600) mutations and microsatellite instability (MSI) [14]. *PTEN* (exons 3–8) and cytosine-phosphate-guanine (CpG) island methylator phenotype (CIMP) status were evaluated in 744 and 489 patients, respectively. Regarding *PTEN*, mutations were detected in 43 out of 744 (5.8%) patients: 33 (76.7%) cases harbored 1 somatic mutation and 10 (23.3%) tumors presented 2 or more mutations. Nine (1.2%) patients harbored both *PIK3CA* and *PTEN* mutations. The presence of a *PTEN* mutation was significantly associated with a right-sided tumor, mucinous histology, high MSI status, *BRAF* mutation, and high CIMP status. Considering cancers with a high MSI status, the association between *PTEN* and *BRAF* mutations remained significant (*p* = 0.019). No significant correlations were found with age, gender, tumor stage, grading, and *KRAS* mutations.

Based on these findings, Day et al. showed an association between the sessile-serrated pathway of CRC development (characterized by high MSI and CIMP statuses, the proximal site of primary tumor, *BRAF* mutation, and *KRAS* wild-type (wt) status) with *PIK3CA* exon 20 and/or *PTEN* mutation [14].

Colakoglu et al. analyzed PTEN expression in 76 primary CRCs showing a negative correlation with young age, female sex, and left-sided tumors [15].

Zhou et al. aimed to determine the association between *PTEN* mutations and MSI status in CRCs by analyzing 11 hereditary nonpolyposis colon cancers (HNPCCs), 32 microsatellite instable sporadic cancers, and 39 microsatellite stable tumors. *PTEN* somatic mutations were found in 18% of HNPCCs and in 13% of microsatellite instable sporadic tumors, whereas no mutations were detected in microsatellite stable CRCs. PTEN expression loss was found in 31% of HNPCCs and 41% of microsatellite instable sporadic CRCs, respectively. Among microsatellite stable CRCs, 17% presented a decreased PTEN expression, but none had a complete expression loss. These findings suggest that PTEN alterations are associated with HNPCC and sporadic microsatellite instable tumors are a consequence of a mismatch repair deficiency [10].

Furthermore, a retrospective analysis investigated the correlation between PTEN expression and clinicopathological factors pairing 69 primary CRCs of patients with corresponding liver metastases with 70 primary CRCs of patients without liver metastases. PTEN expression loss was more frequent in CRCs with liver metastases and showed a significant association with the advanced TNM stage (*p* < 0.01) and lymph node metastasis (*p* < 0.05) [16]. A positive correlation of PTEN expression with histological grade (*p* = 0.006) and distant metastasis (*p* = 0.015) was demonstrated by Lin et al. in 139 CRC patients [17]. Similar results were found by Li et al. showing a positive association between low PTEN expression and tumor size, invasion depth, lymphatic invasion, lymph node metastasis, and higher Dukes staging (*p* < 0.05) in a sample of 327 CRCs [18].

In conclusion, PTEN alterations seem to be more frequently correlated with right-sided tumors, microsatellite instability, *BRAF* mutations, lymph node metastases, and a higher tumor stage.

## 3. PTEN as a Predictive Factor

Monoclonal antibodies directed against the epidermal growth factor receptor (EGFR) clearly revolutionized metastatic CRC (mCRC) treatment, improving clinical response and survival rate, as well as disease control, in addition to tailoring CRC therapy based on tumor molecular characterization.

Adoption of *RAS* and *BRAF* status determination as a crucial decision-making step for mCRC treatment was mainly based on their negative predictive impact toward anti-EGFRs. Those findings deeply affected subsequent research efforts, which were then focused on the identification of additional determinants of benefit.

PTEN loss was explored within putative mechanisms of resistance to EGFR inhibition among *RAS* wt mCRCs. Different mechanisms (i.e., mono- or bi-allelic inactivation, epigenetic silencing), and tumor types (i.e., breast [19], CRC [10], and lung [20] cancers) are known for PTEN loss. PTEN loss assessed using immunohistochemistry (IHC) was suggested to predict trastuzumab resistance in patients with Her2 positive breast cancer [21] and gefitinib in in vitro models [22]. Preclinical data showed the importance of a PTEN/PI3K/AKT pathway determining the CRC cell line sensitivity to cetuximab, and in particular, PTEN loss presents a resistance to cetuximab-induced apoptosis [23]. A first small clinical experience suggested that PTEN loss on primary CRCs could be responsible for cetuximab resistance [24].

In a retrospective study, Loupakis et al. [25] analyzed, by means of IHC, 96 primary tumors and 59 metastases from CRC patients treated with anti-EGFR. The study supported the concept that PTEN expressions may differ in metastases (compared with primary tumors), and that the predictive role for PTEN expression toward anti-EGFRs was only evident when testing the available metastatic samples. Responding patients had significantly more PTEN-positive metastases (36%) compared with those who had PTEN-negative metastases (*p* = 0.007); this translated into a significant difference in progression-free survival (PFS) favoring PTEN positive tumors (hazard ratio (HR) = 0.49; *p* = 0.005). The authors concluded that PTEN loss in metastases deserves further investigation to understand whether it predicts resistance to cetuximab plus irinotecan. Amongst the limitations affecting the significance of this research is that metastatic samples were mostly retrieved from distant lesions resected after a previous conversion therapy. This may have caused a significant selection bias since systemic treatment effects on PTEN expression were not explored.

Laurent-Puig et al. conducted a similar study in advanced mCRC subjects treated with anti-EGFRs, reporting multivariate analyses of shorter overall survival (OS) for *KRAS* wt patients affected by tumors with PTEN loss and *BRAF* mutations [26]. Nevertheless, those results were not further confirmed even if the concept of PTEN expression modulation over time and its difference between primary tumors and metastases has never been prospectively explored.

The most recent and comprehensive studies did not confirm the hypothesis that PTEN alterations are of benefit to predicting anti-EGFRs in CRC [27,28] compared with an initial large meta-analysis [26]. Current research efforts are focused on more refined molecular selection criteria coupled with newly established clinical determinants, such as primary tumor location. Whether a new role for post-trascriptional regulators of PTEN is useful as a predictive marker will be a matter of future exploratory analyses [29].

In addition to the potential role of PTEN status as a biomarker of resistance to anti-EGFR therapies in patients with mCRC, several researches evaluated the impact of PTEN status on the responsiveness to other targeted treatments, including anti-VEGF (vascular endothelial growth factor), drugs targeting the PI3K/AKT/mTOR or the RAS/RAF/MAPK signaling pathways, and poly(ADP-ribose) polymerase (PARP) inhibitors. Concerning anti-angiogenics, contradictory evidence is available on the role of PTEN expression as a response predictor for bevacizumab-based treatments. The rationale supporting the evaluation of PTEN status as a biomarker for bevacizumab-containing regimens is based on the interaction between the PI3K/AKT/mTOR signaling pathway and VEGF expression [30,31,32]. Indeed, mTORC1 (mammalian target of rapamycin complex 1) modulates hypoxia-inducible factor 1 alpha (HIF1α) transcription, which in turn increases VEGF expression [33,34]. Price et al. hypothesized that in the absence of PTEN, which usually counteracts PI3K, aberrant PI3K activity upregulates HIF1α, resulting in increased VEGF expression [12]. Therefore, bevacizumab-based regimens might be more active in patients affected by mCRC with a loss of PTEN expression.

In 2012, a retrospective analysis compared the PTEN expression (assessed by means of IHC) of 34 tumor samples from patients affected by mCRC treated with bevacizumab-based regimens with treatment activity [35]. No statistically significant differences were found between the response rate and different expression levels of PTEN (*p* = 0.832).

In 2013, Price et al. performed a post hoc analysis on tumor samples of patients treated within the AGITG MAX trial [12]. The AGITG MAX trial was a randomized phase III trial, which compared capecitabine +/− bevacizumab (+/− mitomycin C) in the first-line treatment of patients affected by mCRC. The post hoc analysis involved 302 (64.1%) patients and assessed *PTEN* expression by means of a copy number assay, aiming to evaluate the predictive impact of *PTEN* loss in patients receiving bevacizumab. *PTEN* loss was reported in 38.7% of patients. The addition of bevacizumab did not significantly increase the response rate (RR), PFS, and OS among patients with a loss of *PTEN* expression compared with those without (*p*-value for the interaction between *PTEN* expression and treatment = 0.36, 0.26, and 0.35, respectively). Thus, the authors concluded that the *PTEN* loss assessed using a copy number assay was not predictive for bevacizumab combined with capecitabine in a first-line mCRC treatment.

More recently, another retrospective analysis conducted on 42 patients with mCRC receiving bevacizumab-containing combinations in a first- or second-line treatment showed that a loss of PTEN protein expression in secondary tumor tissue samples was significantly associated with the treatment response (*p* = 0.02; *p*-value adjusted for prognostic factors = 0.006) [36]. However, this correlation was not confirmed in the survival analysis.

*PIK3CA* mutations and PTEN loss of function have been suggested to be strong predictors for serine-threonine kinase mammalian target of rapamycin (mTOR) inhibitor sensitivity [37,38]. Two phase I trials assessing the selective mTOR inhibitor everolimus, administered as a monotherapy in advanced solid tumors, documented two partial responses against mCRC [39,40]. However, such preliminary evidence of activity was not subsequently confirmed, as two phase II trials evaluating everolimus in refractory mCRC did not report any objective responses [41]. Interestingly, the PI3K/AKT/mTOR signaling pathway has a potential role in modulating the effect of bevacizumab. Besides inhibiting cell cycles, mTOR inhibition may interfere with angiogenesis suppressing the expression of hypoxia-inducible factors (HIFs) [42]. Moreover, hypoxia caused by anti-angiogenics may cause treatment resistance and tumor progression due to a HIFs increase, which activates the genes involved in cell survival, metastasis, and drug resistance [43,44]. Therefore, it has been hypothesized that adding mTOR inhibitors to antiangiogenics might preserve the benefits of impairing angiogenesis, at the same time avoiding negative impacts of increased hypoxia on the tumor biology, which leads to acquired aggressiveness [45]. Given the postulated PI3K/AKT/mTOR pathway role modulating anti-VEGF activity, mTOR inhibitors were tested in combination with bevacizumab-containing regimens in mCRC. A phase I/II trial evaluated the safety and efficacy of everolimus in combination with mFOLFOX6 plus bevacizumab as a first-line treatment in mCRC patients. In a post hoc analysis, the response rate was assessed according to PTEN expression [46]. The overall response rate was 53% in the whole population, 40% in patients with PTEN above the threshold, and 86% in patients with PTEN below the threshold (*p* = 0.03).

Based on preclinical data suggesting that the combination of temsirolimus and bevacizumab may increase antitumor activity and re-sensitize cells to anthracyclines [47], a phase I study assessing the activity of bevacizumab and temsirolimus plus liposomal doxorubicin in patients with advanced malignancies was conducted [48]. The trial enrolled 136 patients, including 17 patients affected by mCRCs. The response rate was significantly higher in patients with a *PIK3CA* mutation and/or a *PTEN* mutation or loss of expression (*p* = 0.018).

Beyond anti-EGFRs, PTEN was tested as a response predictor to other drugs targeting the RAS/RAF/MAPK signaling pathway. In an open-label phase I/II study, assessing the safety and activity of the combination of BRAF and MEK inhibitors (dabrafenib plus trametinib) in patients affected by *BRAF* V600-mutant mCRCs, archival tissue samples were analyzed for PTEN status [49]. PTEN (assessed using IHC) was evaluated in 20 out of 43 enrolled patients, and a loss of expression was identified in 4 patients. All patients with a PTEN loss of expression achieved a shrinkage of the target lesions; however, no difference in PFS was observed according to PTEN status.

Recently, Pishvaian et al. published the results of a single-arm, open-label, phase II study that investigated the activity of veliparib plus temozolomide in patients with refractory mCRC [50]. The combination was well tolerated and active, with a disease control rate of 24%, a PFS of 1.8 months, and an OS of 6.6 months. IHC for PTEN was performed on archival tumor samples and PTEN expression levels were compared with the treatment activity based on pre-clinical evidence of altered homologous recombination displayed by tumors without PTEN expression [51,52] and on the predictive role of PTEN loss for PARP inhibitors in endometrial cancers [53,54]. The absence of PTEN expression was not associated with the disease control rate; thus, the study failed to demonstrate a correlation between PTEN loss and a response to PARP inhibitors.

Data concerning the role of PTEN deficiency as a predictive marker in mCRC receiving target treatment (Table 1) are contradictory and should be considered exploratory. The main limit of studies assessing PTEN predictive values is found in the determination of tumor PTEN status. As previously described, PTEN expression may be lost by both genomic and non-genomic mechanisms; moreover, PTEN-positive tumors may display an impaired PTEN function. In order to assess the predictive role of PTEN, the tumor PTEN status should be evaluated using both protein quantification and DNA sequencing, and PTEN phosphatase activity should also be quantified.

Up to now, a comprehensive assessment of PTEN status represents a challenge. Therefore, the role of PTEN as a predictor of a response to target treatments cannot be established yet and further studies are warranted.

## 4. PTEN as a Target

**Figure 2:**
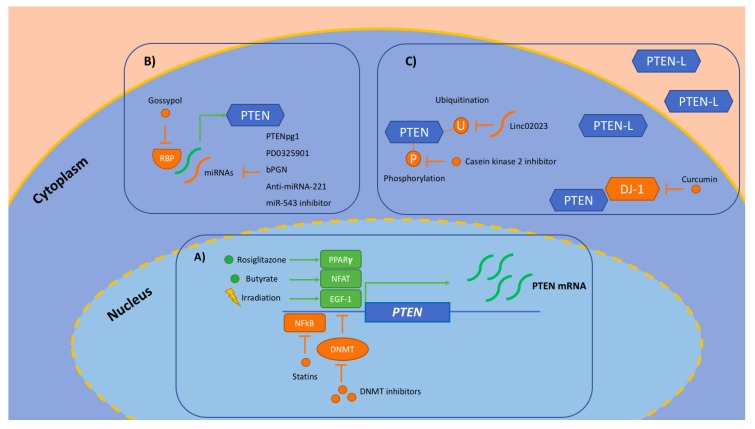
PTEN as a target. Strategies aiming at restoring *PTEN* onco-suppressor functions that have been hypothesized and are currently under evaluation in early phases of research in preclinical settings. (**A**) Transcriptional level: increase of *PTEN* transcription achieved by removing epigenetic silencing via DNMT inhibitors, or by modifying (increasing or reducing) exposure to transcription factors. (**B**) Post-transcriptional level: enhanced PTEN translation via the modulation of regulatory miRNAs and RBP. (**C**) Post-translational level: modulation of PTEN modifications, which regulate PTEN activity, conformation and subcellular compartmentalization, and protein–protein interactions. EGR-1: early growth response protein 1. DNMT: DNA methyltransferase. miRNA: microRNA. NFAT: nuclear factor of activated T-cells. NF-κB: nuclear factor kappa-light-chain-enhancer of activated B cells. PPARγ: peroxisome proliferator-activated receptor gamma. PTEN: Phosphatase and tensin homolog. RBP: RNA-binding protein.

Restoration of PTEN expression and function exerts direct antitumoral activity, which reduces tumor cell proliferation, invasiveness, and at the same time, stimulates apoptosis sensitizing cells to cytotoxicity, target agents, immunotherapies, and radiation [13]. Given diverse mechanisms that lead to PTEN inhibition in CRC, several strategies aiming to restore oncosuppressor functions have been hypothesized and are currently under evaluation in the early phases of preclinical research (Figure 2; Table 2) [56].

Increased PTEN function can be pursued through potentiating *PTEN* transcription. *PTEN* transcription can be achieved by removing an epigenetic block or by modifying (increasing/decreasing) the exposure to activating or inhibiting transcription factors [56]. The epigenetic silencing of *PTEN* transcription is due to gene promoter or histone methylation [57]. Epigenetic target treatments are emerging as potential options for solid tumors. DNA methyltransferase inhibitors remove methyl groups from DNA, causing the demethylation of DNA. Early studies reported the activity and safety of decitabine in combination with panitumumab in *KRAS* wt mCRC patients previously treated with cetuximab [58]. Decitabine proved to be safe when administered via hepatic arterial infusion in CRC patients with unresectable predominant liver metastases [59]. Preliminary data indicate that treatment with DNA demethylating drugs upregulates specific immune gene sets [60], displaying an immune stimulatory role. The combination of epigenetic modulators with immunotherapy are further investigated in microsatellite stable mCRC based on the postulated ability to enhance the response to immunotherapy. Hypomethylating agents (azacitidine, decitabine, guadecitidine) are currently under evaluation in clinical settings for CRC treatment in combination with chemotherapy (NCT01193517, NCT01896856) or with immunotherapic drugs: pembrolizumab (NCT02260440, NCT0251217, NCT02959437), nivolumab (NCT03576963), durvalumab (NCT02811497), and the allogeneic CRC cell vaccine (GVAX) (NCT01966289). None of the trials planned to evaluate treatment effects on modulating PTEN expression. However, such an analysis would be of great interest. As previously stated, *PTEN* transcription is regulated by transcription factors. Such molecules can bind the *PTEN* promoter and activate or inhibit gene transcription. Some of these transcription factors can be pharmacologically stimulated: peroxisome proliferator-activated receptor gamma, PPARγ (via rosiglitazone); early growth response protein 1, EGR-1 (via irradiation); nuclear factor of activated T-cells, NFAT (through butyrate, a fatty acid produced by colonic microbiota fermentation). On the contrary, the inhibiting transcription factor NF-κB (nuclear factor kappa-light-chain-enhancer of activated B cells) can be repressed through statins or selective inhibitors [56].

At the post-transcriptional level, PTEN expression can be impaired by microRNAs (miRNAs) or RNA-binding protein (RBP). miRNAs are short non-coding RNAs that bind mRNAs, causing translation inhibition or transcript degradation, which ultimately results in a loss of PTEN expression and activation of the PI3K/Akt signaling cascade. Several miRNAs [61,62] and a complex of RBP known as Musashi-1/2 [63], targeting PTEN in CRC, have been identified. Therefore, modulation of those regulatory RNAs and RNA-binding proteins represent a therapeutic strategy aiming at restoring PTEN translation and expression, exploiting its antitumor activity and increasing cellular drug sensitivity. Concerning such a strategy, some in vitro evidence found regarding human CRC cell lines are available. Notably, an anti-miRNA-221 was shown to increase PTEN expression, sensitizing CRC cells to radiation [64]. Butylcycloheptyl prodiginine (bPGN) is a prodiginine-type agent able to suppress oncomir miR-21 and consequently cellular growth in CRC lines through the inhibition of Dicer-mediated processing of pre-miR-21 [65]. The administration of a miR-543 inhibitor was shown to reverse the chemoresistance of 5-fluorouracil (5-FU) obtained by this oncomir through a reduction of PTEN expression, enhancing cellular sensitivity to 5-FU [66]. PD0325901 (a MEK inhibitor) caused PTEN upregulation by suppressing the miR-17-92 cluster [67]. miRNAs can be saturated by the PTENpg1 transcript, a long, noncoding RNA (lncRNA) transcripted by the PTEN pseudogene (*PTENpg1*) [68]. Gossypol (a natural phenol extracted from cottonseed) showed inhibiting features toward Musashi-1/2 proteins and demonstrated antitumoral activity in a xenograft model [63]. Phase I/II clinical trials showed no activity in prostate cancer and non-small-cell lung cancer [69,70].

Several PTEN isoforms originating from different start codon translations have been identified. Of those, PTEN-L retains a secretion ability and exerts paracrine function interfering with intracellular signaling and survival of the surrounding cells. PTEN-L was shown to counteract the PI3K/Akt pathway, leading to cell death, both in vitro and in vivo (through intraperitoneal infusion in xenograft models) [71]. Interestingly, this isoform has been engineered to increase cell-mediated delivery [72].

Post-translational modifications (including phosphorylation, oxidation, S-nitrosylation, S-sulfydration, acetylation, methylation, ubiquitinylation, sumoylation, and ribosylation) at specific aminoacidic residues can directly modulate PTEN catalytic or binding activity, or PTEN conformation and subcellular compartmentalization, subsequently impacting PTEN function [13,56]. Reverting those post-translational modifications or targeting enzymes that are involved could be effective at restoring PTEN function in PTEN positive neoplasms [56]. For example, in vitro exposure of CRC lines to a casein kinase 2 (a serine/threonine kinase that phosphorylates PTEN, causing repression of its catalytic activity) inhibitor caused reduced cell growth and invasiveness [73]. The lncRNA Linc02023 was shown to impair PTEN ubiquitination and subsequent degradation, positively correlating with PTEN expression, inhibiting CRC cell proliferation and in vitro and in vivo survival [74]. This molecule could represent a novel therapeutic agent that restores the PTEN tumor suppressor function.

Finally, PTEN exerts pleiotropic functions by being included in multiprotein complexes. Several proteins interact with PTEN, regulating (both positively and negatively) tumor suppressing functions [75,76,77]. Therefore, an intriguing strategy to modulating PTEN activity is to target protein–protein interactions. Curcumin, a phenolic agent derived from vegetables, showed in vitro antitumor activity by inhibiting proliferation and promoting apoptosis in CRC cell lines via the downregulation of DJ-1 (a PTEN negative modulator) and consequently promoting PTEN function [78]. A ribonuclease inhibitor is a cytosolic protein that inactivates ribonucleases via high affinity binding. In CRCs, the cell line upregulation of ribonuclease inhibitors was shown to stimulate PTEN expression leading to PI3K/Akt pathway suppression [79].

In conclusion, restoring PTEN expression, and ultimately activity, could have therapeutic implications for CRC patients. Targeting PTEN is an intriguing field of research to explore CRC treatment strategies, although challenging to achieve.

## 5. Future Perspectives

In the future, two main settings should be discriminated to target PTEN according to their gene status. First, *PTEN* mutated neoplasms—characterized by a loss of expression due to a genomic aberration, such as mutations and copy number variation—and second, *PTEN* wt neoplasms—characterized by a loss of PTEN expression that could derive from epigenetic, transcriptional, or translational alterations, or by a loss of PTEN function due to post-translational modulation.

In *PTEN* wt neoplasms, two main strategies could be hypothesized according to the level of PTEN regulation impairment. First, in PTEN negative cells, due to transcriptional or translational aberration that leads to protein loss, the therapeutic approach should aim at restoring PTEN expression; whereas in PTEN positive cells, in which the protein is present, although not retaining functions due to post-translational alterations, the strategy should aim at restoring PTEN function that was aberrantly inhibited.

Concerning *PTEN* mutated neoplasms, due to a genomic aberration, such as mutations and copy number variations, loss of PTEN expression could act as a response predictor for treatments targeting the PI3K/Akt pathway. Evidence concerning such a role are contradictory. Moreover, since cells lacking nuclear PTEN are hypersensitive to DNA damage because of impaired homologous recombination, this defect could sensitize tumor cells to PARP inhibitors [52,53,80]. *PTEN* mutated neoplasms might be responsive to PARP inhibitors and *PTEN* genomic status could be exploited as a predictor of the response to these agents [13].

In the meantime, the National Cancer Institute (NCI) has developed the NCI-MATCH (Molecular Analysis for Therapy Choice) trial (ClinicalTrials.gov Identifier: NCT02465060), an umbrella precision medicine cancer treatment clinical trial. In this ongoing study, patients with advanced solid tumors (including CRC), lymphomas, or myeloma, are assigned to receive treatment based on genetic tumor changes identified by genomic sequencing and other tests. Patients whose tumors have genetic changes that match one of the trial treatments may be enrolled if they meet other eligibility criteria. The trial aims to determine whether cancer treatment based on specific genetic changes is effective, regardless of cancer type. The primary end-point of NCI-MATCH trial is the response rate. Treatments will be considered promising if at least 16% of patients in an arm reach a complete or partial response.

Among treatment arms that are open and enrolling patients, Z1G and Z1H allow for the enrolment of patients with tumors harboring a *PTEN* mutation or those characterized by a PTEN loss to receive copanlisib, a PI3K inhibitor.

## 6. Conclusions

This review presented available data regarding the role of PTEN as a predictive factor for standard mCRC therapy, in particular for anti-EGFR, and as a possible target for future innovative treatments. Although PTEN is well-known tumor suppressor gene, known since the 1990s, it has not yet entered into full clinical practice. Its role as a target is certainly the most intriguing and innovative aspect. Although targeting PTEN is a difficult challenge, it might represent an extra step toward the customization of treatments in mCRC.

## Figures and Tables

**Table 1:** Clinical evidence for PTEN as a predictor of the response to target treatments.

	Study	No. of Patients	Treatment	PTEN Assessment	RR	PFS	OS
**Frattini et al. 2007 [24]**	Prospective	27	Cet-based	IHC	PTEN+ vs. PTEN− 62.5% vs. 0% (*p* > 0.001)	-	-
**Loupakis et al. 2009 [25]**	Retrospective	59	Iri + Cet	IHC	PTEN+ vs. PTEN− Higher RR (*p* = 0.007)	PTEN+ vs. PTEN- 4.7 vs. 3.3 m (HR = 0.49; *p* = 0.005)	-
**Laurent-Puig et al. 2009 [26]**	Retrospective	162	Cet-based	IHC	-	-	PTEN- associated with shorter OS (*p* = 0.013)
**Therkildsen et al. 2014 [55]**	Meta-analysis	100 (9 studies)	Anti-EGFR based	Protein expression (7 studies) Mutational status (2 studies)	PTEN- Odds Ratio = 0.41 (95%CI = 0.20–0.85)	PTEN- associated with shorter PFS (HR 1.88, 95%CI = 1.35–2.61)	PTEN- associated with shorter OS (HR = 2.09, 95%CI = 1.36–3.19)
**Karapetis et al. 2014 [28]**	CO.17 trial Prespecified subgroup analysis	205	Cet	IHC	PTEN+ vs. PTEN− 21% vs. 15%	-	No association between PTEN status and OS Among PTEN+ OS 9.9 vs. 5.4 months for Cet vs. BSC (HR = 0.66; *p* = 0.32)
**Agoston et al. 2016 [27]**	Retrospective	55	Anti-EGFR based	IHC	-	-	No association between PTEN status and OS
**Kara et al. 2012 [35]**	Retrospective	34	Bev based	IHC	PTEN+ vs. PTEN− *p* = 0.832	-	PTEN+ vs. PTEN− *p* = 0.6
**Price et al. 2013 [12]**	AGITG MAX trial, *post hoc* analysis	302	Bev based	CNV	*PTEN*+ vs. *PTEN*− *p* = 0.36	*PTEN*+ vs. *PTEN*− *p* = 0.26	*PTEN*+ vs. *PTEN*− *p* = 0.35
**Sclafani et al. 2015 [36]**	Retrospective	42	Bev based	IHC	PTEN− vs. PTEN+ 71.4% vs. 32.1% *p* = 0.02	PTEN− vs. PTEN+ 9.2 vs. 8.7 months *p* = 0.968	PTEN− vs. PTEN+ 21.1 vs. 17.3 months *p* = 0.628
**Weldone Gilcrease et al. 2019 [46]**	Post hoc analysis (phase I/II)	24	Eve+mFOLFOX6-Bev	IHC	PTEN+ vs. PTEN− 40% vs. 86% *p* = 0.03	-	-
**Moroney J et al. 2012 [48]**	Prospective (phase I)	136 (including 17 with mCRC)	Tem+Bev+liposomial doxo	PCR and IHC	PIK3CA MT and/or PTEN loss/MT vs. WT 39% vs. 16%, *p* = 0.018		
**Corcoran RB et al. 2015 [49]**	Prospective (phase I/II)	19	Dabrafenib+ Trametinib	IHC	PTEN− vs. PTEN+ 21% vs. 0%	PTEN− vs. PTEN+ 3.48 vs. 3.61 months *p* = 0.35	-
**Pishvaian et al. 2018 [50]**	Prospective (phase II)	49	Veli+Temo	IHC	PTEN− vs. PTEN+ 13.3% vs. 21.1%	PTEN− vs. PTEN+ 1.7 vs. 1.8 months	PTEN- vs. PTEN+ 6.2 vs. 6.3 months

Table 1 summarizes clinical evidences on PTEN as a predictive factor. Bev: bevacizumab; BSC: best supportive care; Cet: cetuximab; Doxo: doxorubicin; Eve: everolimus; IHC: immunohistochemistry; Iri: irinotecan; m: months; MT: mutation; N: number; OS: overall survival; PFS: progression free survival; RR: response rate; Temo: temozolomide; Tems: temsirolimus; Veli: veliparib; WT: wild type.

**Table 2:** PTEN as a target. Strategies aiming at restoring PTEN onco-suppressor functions that have been hypothesized and are currently under evaluation in early phases of research in preclinical settings.

Level	Strategy	Agents	Evidences	Reference
Transcriptional level	Removing epigenetic inhibition	DNA methyltransferase inhibitors	Decitabine proved to be safe and active in combination with panitumumab in KRAS wt mCRC patients previously treated with cetuximab. Decitabine proved to be safe when administered by hepatic arterial infusion in liver limited mCRC patients.	[58] [59]
	Increasing exposure to activating transcription factors	Rosiglitazone Irradiation Butyrate	Some transcription factors can be pharmacologically stimulated: PPARγ (via rosiglitazone), EGR-1 (via irradiation), NFAT (via butyrate).	[56]
	Reducing exposure to inhibiting transcription factors	Statins NF-κB selective inhibitors	The inhibiting transcription factor NF-κB can be repressed through statins or selective inhibitors.	[56]
Post-transcriptional level	Inhibiting miRNAs and RNA binding proteins	Anti-miRNA-221	Anti-miRNA-221 showed to increase PTEN expression, sensitizing CRC cells to radiation.	[64]
		Butylcycloheptyl prodiginine	Butylcycloheptyl prodiginine showed to suppress miR-21 and consequently cellular growth in CRC lines.	[65]
		miR-543 inhibitor	A miR-543 inhibitor proved to reverse chemoresistance to 5-fluorouracil (5-FU), obtained by this oncomir through reduction of PTEN expression, enhancing cellular sensitivity to 5-FU.	[66]
		PD0325901	PD0325901 (a MEK inhibitor) proved to upregulate PTEN by suppressing miR-17-92 cluster.	[67]
		PTENpg1	PTENpg1, a long, non-coding RNA transcripted by the PTEN pseudogene (*PTENpg1*) was shown to saturate miRNAs.	[68]
		Gossypol	Gossypol showed to inhibit Musashi-1/2 proteins and demonstrated antitumoral activity in a xenograft model.	[63]
Post-translational level	Targeting enzymes involved in post-translational modification or reverting post-translational modification	Casein kinase 2 inhibitor	Inhibitor of casein kinase 2 (a serine/threonine kinase, which phosphorylates PTEN, causing repression of its catalytic activity) showed to reduce cell growth and invasiveness in CRC lines.	[73]
		Linc02023	Linc02023 (a long non coding RNA) was shown to impair PTEN ubiquitination and subsequent degradation, inhibiting CRC cell proliferation and in vitro and in vivo survival.	[74]
	Paracrine function	PTEN-L	PTEN-L, an PTEN isoform with a paracrine function, showed to counteract the PI3K/Akt pathway both in vitro and in vivo (through intraperitoneal infusion in xenograft models).	[71]
	Target protein–protein interaction	Curcumin	Curcumin was shown to inhibit proliferation and promote apoptosis via the downregulation of DJ-1 (a PTEN negative modulator) in CRC cell lines.	[78]
		Ribonuclease inhibitor	Upregulation of a ribonuclease inhibitor was shown to stimulate PTEN expression, leading to PI3K/Akt pathway suppression in CRC cell lines.	[79]
